# Brain neurons internalise polymeric micron-sized capsules: Insights from *in vitro* and *in vivo* studies

**DOI:** 10.1016/j.mtbio.2025.101493

**Published:** 2025-01-21

**Authors:** Olga Kopach, Olga A. Sindeeva, Kaiyu Zheng, Eleanor McGowan, Gleb B. Sukhorukov, Dmitri A. Rusakov

**Affiliations:** a Department of Clinical and Experimental Epilepsy, UCL Queen Square Institute of Neurology, University College London, London WC1N 3BG, UK; b Neuroscience and Cell Biology Research Institute, City St George's University of London, Cranmer Terrace, London SW17 0RE, UK; c School of Engineering and Materials Science, Queen Mary University of London, Mile End Road, London E1 4NS, UK

**Keywords:** Polyelectrolyte microcapsules, Brain neurons, Internalisation, Intracellular delivery, Brain targeting

## Abstract

Nanoengineered encapsulation presents a promising strategy for targeted drug delivery to specific regions in the body. While polyelectrolyte-based biodegradable microcapsules can achieve highly localised drug release in tissues and cell cultures, delivering drugs to intracellular sites in the brain remains a significant challenge. In this study, we utilized advanced imaging techniques, both *in vitro* and *in vivo*, to investigate whether brain neurons can internalise polyelectrolyte-based microcapsules designed for drug delivery. High-resolution live-cell imaging revealed that differentiating N2A cells actively internalise microcapsules, often incorporating multiple capsules per cell. Likewise, primary hippocampal and cortical neurons were observed to effectively internalise polymeric microcapsules. In the intact brain, multiplexed two-photon excitation imaging *in vivo* confirmed the internalisation of microcapsules by cortical neurons following delivery to the somatosensory brain region. This internalisation was time-dependent, correlated with particle size and mediated by a macropinocytosis mechanism that appears to bypass lysosomal formation. Importantly, the presence of internalised microcapsules did not impair neuronal function, as neurons maintained normal firing activity and action potential characteristics. Furthermore, no adverse effects were observed after a week of microcapsule presence in the mouse brain. Our findings indicate that polymeric microcapsules are effective and safe carriers for intracellular drug delivery to brain neurons, providing a targeted approach with potential therapeutic applications.

## Graphical abstract

**Figure:**
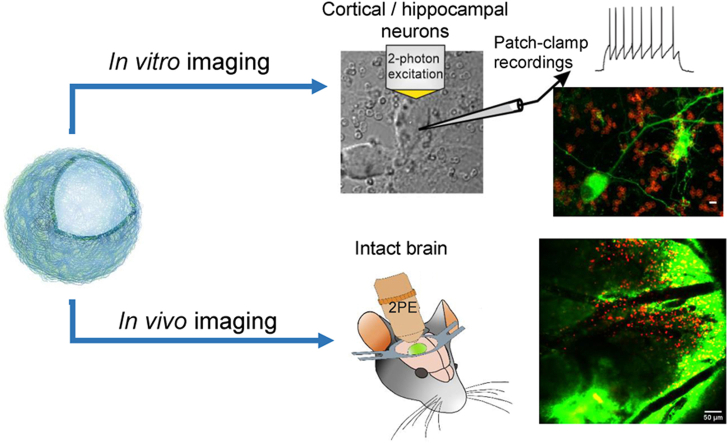

## 1 Introduction

Drug delivery using nanoengineered encapsulation offers significant advantages in the biomedical field, particularly in achieving precision targeting. Encapsulation enhances the stability of bioactive compounds, ensures sustained and controlled drug release, reduces off-target effects, and minimises adverse effects of treatments (for review, see Refs. [1,2]). Despite its numerous advantages, targeted delivery to the central nervous system remains a significant challenge and highly in demand. Effective targeting of the brain requires drug delivery approaches to meet multifaceted demands, accounting for the complex structure of brain tissue. The unique morphology of neuronal cells poses additional challenges for precision targeting, as these cells feature relatively small cell bodies, intricate dendritic trees, and axons that often extend far from the somata across the tissue. Ensuring adequate bioavailability and pharmacokinetics for compounds with intracellular sites of action is even more difficult. These compounds must not only penetrate the brain's protective barriers but also enter neurons to reach their intracellular targets, further complicating the development of effective delivery systems.

Various types of nanoparticles have been probed for targeted drug delivery to the brain, particularly in cancer therapy, with some approaches advancing to clinical trials. Among these are liposomes, polymeric organic and inorganic nanoparticles, which have facilitated the selective delivery of anticancer drugs to tumour cells while minimising damage to healthy cells [[3], [4], [5], [6], [7]]. Beyond the anticancer treatments, nanoparticles have been utilized successfully for the delivery of gene materials, such as cDNA, RNA, oligonucleotides, proteins, or CRISPR-Cas9 complex [[8], [9], [10], [11], [12]]. Nanocarrier approaches have been proven to enhance safety and reduce immunogenicity and off-target effects. To enhance the permeability of nanoparticles, nanomaterial compositions have undergone continuous optimisation. For instance, efforts have focused on improving nanoparticle hydrophilicity and enhancing their interactions with cell surface receptors. This has been achieved by incorporating non-charged polymers, surfactants, or polymer coatings designed to degrade *in vivo* [[13], [14], [15], [16]]. *In vitro* studies have demonstrated that nanoparticles can enter and accumulate in cells in high quantities through direct fusion with the plasma membrane [17] or via endocytosis [14,18]. While endocytosis is considered the principal route for nanoparticle uptake, various cellular internalisation mechanisms and pathways can be involved, depending on the design and surface functionalisation of the particles [17,19,20].

Despite the intracellular internalisation of nanoparticles by various cells of non-neuronal types being well documented (for review, see Refs. [21,22]), studies in nerve cells remain limited. It has been demonstrated the uptake of liposomes by astrocytes and microglia [23,24] and the internalisation of nanoparticles by dorsal root ganglion cells and microglia in the peripheral nervous system [25,26] and by brain neurons *in vivo* [27,28]. However, it remains unclear whether brain neurons can internalise microcarriers (above micron size), which offer higher drug-loading capacities than nanocarriers and hold potential advantages for drug delivery.

Our previous studies have shown the significant therapeutic potential of polyelectrolyte-based polymeric microcapsules (1–3 μm in size) for localised drug delivery to peripheral nerves [29] and guided neurite outgrowth by hippocampal neurons *in vitro* [30]. The layer-by-layer (LbL)-fabricated polyelectrolyte-based polymeric microcapsules present a multifaceted drug delivery platform, versatile and relatively simply fabricated, with multiple possibilities for their high-performance functionalisation and controlled release [[31], [32], [33], [34], [35], [36]]. However, whether neurons can efficiently internalise such relatively large particles remains unknown. In this study, we combined live-cell two-photon excitation imaging, both *in vitro* and *in vivo*, with single-cell electrophysiology to investigate whether brain neurons can internalise LbL-fabricated polyelectrolyte-based microcapsules while maintaining normal physiological function. Our observations offer valuable insights for intracellular neuronal delivery, advancing pharmacological and genetic-based therapies.

## 2 Materials and methods

### 2.1 Fabrication of LbL-microcapsules

Polyelectrolyte-based microcapsules were fabricated using the LbL-assembly technique, as we described in detail previously [29,30,37]. Various fabrication variants were made in terms of material compositions and particle sizes. For the fabrication process, the first step was preparing calcium carbonate (CaCO_3_) microspheres as templates for microcapsule synthesis. For this, equal volumes (0.65 ml) of calcium chloride and sodium carbonate water solutions (1 M) were combined under constant stirring in 3 ml of deionized water. The size of the resulting microspheres was determined by the stirring speed during synthesis. The microcapsule shell was obtained at the next step by sequential precipitation of two oppositely charged polyelectrolytes: poly(allylamine hydrochloride) (PAH, MW = 17.5 kDa) and poly(4-styrene sulfonate) sodium salt (PSS, MW = 70 kDa). Both polyelectrolytes were adsorbed from 2 mg/ml solutions in 0.15 M NaCl. The microcapsule composition consisted of 4 bilayers [PAH/PSS]_4_. PAH-FITC (TRITC) or PAH-RITC conjugates were used to synthesise the third bilayer for the fluorescent properties of the microcapsules. Biodegradable shells were assembled of 3 bilayers of positively charged poly-L-arginine (PArg, MW = 70 kDa) and oppositely charged dextran sulphate sodium salt (DS, MW = 70 kDa) with the resulting shell structure [PArg/DS]_3_. DS in the second bilayer was replaced by a fluorescently labelled polymer (commercially available DS-FITC or TRITC) for the visualisation of microcapsules. Biodegradable polyelectrolytes were deposited on calcium carbonate microparticles from 2 mg/ml solutions in 0.15 M NaCl. In the final step, the CaCO_3_ cores were dissolved in 0.2 M EDTA (pH 6.5 with NaOH).

The suspension of microcapsules in ddH_2_O was stored at 4 °C (a total sample volume of 2 ml) and resuspended before applying to neuronal cultures or for brain injections *in vivo*. Polyelectrolytes and other chemicals were purchased from Sigma-Aldrich (UK).

### 2.2 Characterisation of microcapsules – scanning electron microscopy, confocal laser scanning, and two-photon excitation (2 PE) imaging

**Fig. 1:**
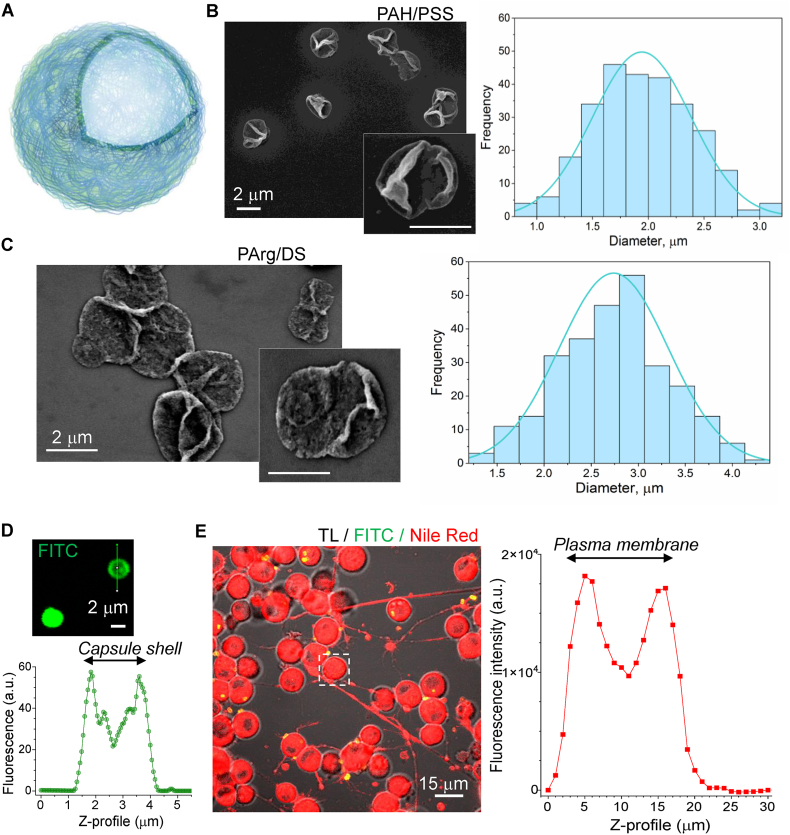
Polyelectrolyte-based microcapsules and experimental design to visualise intracellular microcapsules using high-resolution two-photon excitation (2 PE) imaging in live cells. (A) A schematic illustration of the microcapsule structure. (B) A typical SEM image of a dispersed suspension of PAH/PSS-based microcapsules (left) and analysis of particle dimension distribution for these fabrications (right plots). Scale bar: 2 μm. (C) Same as in B but for the PArg/DS-based microcapsules. Scale bar: 2 μm. (D) Representative 2 PE image of PAH/PSS-based microcapsules at high resolution (FITC, green channel) and fluorescent signal profile of a single microcapsule (shown on the image). Note that a microcapsule size diameter is counted as two peaks of fluorescence signal for the capsule shell. (E) Merged 2 PE image of differentiating N2A cells labelled with a lipophilic dye Nile Red (red channel) in a transmitted light channel (TL) in the presence of FITC-conjugated microcapsules (green channel). The plot demonstrates the fluorescent profile of membrane staining analysed for an individual cell (dotted square on the image) across various focal planes scanned (Z-depth).

Scanning electron microscopy (SEM) images of the fabricated microcapsules were obtained with a VEGA 3LM, TESCAN microscope (Czech Republic, Brno). For this, a droplet of diluted and washed water suspension of microcapsules was plated on a flat silicon substrate attached to a sample holder. Thin gold film (approximately 5 nm thickness) was applied via a rotary-pumped Emitech K350 (Emitech Ltd, England) sputter coater. SEM was carried out at an accelerating voltage of 15 kV and a working distance of 8–10 mm. The capsule size was measured directly from SEM images (Fig. 1B–C) using ImageJ software and calculated as the average diameter at the skeletonised boundary, with the sample size of at least 250 capsules per condition. The potential analyser Zetasizer Malvern Nano ZS (Malvern Panalytical, United Kingdom) was used to measure the zeta potential of the microcapsules.

A confocal laser scanning microscope (CLSM) Leica TCS SP8 X (Leica, Germany) was used for encapsulated RITC visualisation (air objective 20×/0.70 NA). The excitation laser wavelength was 561 nm, and the emission bandpass filter was 580–630 nm.

A dispersed suspension of microcapsules was visualised with two-photon excitation (2 PE) microscopy using a Radiance 2000 imaging system (Zeiss-Bio-Rad) or a Femtonics imaging system Femto-2D optically linked to a Ti: Sapphire Mai-Tai femtosecond pulse laser (SpectraPhysics-Newport). Various digital zooms were used with the appropriate emission filters for imaging the microcapsule-conjugated fluorescence at 2 PE excitation λ_x_^2PE^ = 800 nm for FITC and λ_x_^2PE^ = 820 nm for TRITC.

### 2.3 Neuroblastoma N2A cells

Cultured N2A cells were maintained as we described earlier [38,39]. In brief, cells were cultured in Dulbecco's modified Eagle medium (DMEM, Invitrogen, Carlsbad, CA, USA), containing high glucose and 2 mM L-glutamine, supplemented with 10 % foetal bovine serum (FBS), 2 % penicillin-streptomycin, and 1 % non-essential amino acids at 37 °C. For differentiation of the N2A cells to neuronal phenotype, the culturing medium was a low serum DMEM (2 % FBS instead of 10 %). The cells were harvested using 0.05 % trypsin–EDTA (Gibco, USA) for 5–10 min, washed and plated on glass coverslips. Cell cultures were maintained until used. At least three independent cell culture preparations were tested.

### 2.4 Cytotoxicity assay

Cytotoxicity assessment was conducted using a common MTT assay to test the impact of microcapsules on cell viability. For the test, N2A cells were plated on a 96-well plate (10 × 10^3^ cells/well) and incubated overnight (37 °C, 5 % CO_2_). Microcapsules, in a volume of 100 μl, were resuspended in a fresh growth medium and added to N2A cells on the surface of the plates. The microcapsules were added in ratios of 5, 10, 15, 20, 25, 30, and 35 microcapsules per cell, and the mixture was carefully agitated to ensure a homogenous distribution. The cells were incubated with the microcapsules for 24 h (37 °C, 5 % CO_2_). After the incubation, the medium was replaced with 100 μl of fresh medium containing 10 % MTT stock solution (5 mg/ml in DPBS buffer) and incubated for the next 3 h (37 °C, 5 % CO_2_). Afterwards, the culture medium was replaced with 100 μl of DMSO solution and incubated for 15 min using a thermoshaker (TS-100 Biosan, 37 °C, 300 RPM) for dissolving formazan crystals. The absorbance was measured using a spectrophotometer ClarioSTAR Plus (BMG Labtech, Ortenberg, Germany) at 554 nm wavelength. There were a total of 7 repetitions performed for at least two different preparations tested.

### 2.5 Primary neuronal cultures

Different primary cultures were made to test the microcapsule uptake by various subtypes of brain neurons. We prepared hippocampal neuronal cultures or hippocampal neuronal-astrocytic co-cultures and cortical cultures. Hippocampal neurons or cortical neurons, as appropriate, were isolated from the Sprague-Dawley rat pups (P0 to P2 day old) or C57BL/6J mouse pups (Charles River Laboratories) in accordance with the European Commission Directive (86/609/EEC) and the United Kingdom Home Office (Scientific Procedures) Act (1986). Neurons were cultured in a Neurobasal A/B27-based medium at 37 °C in a humidified atmosphere containing 95 % O_2_ and 5 % CO_2_; for neuronal-astrocytic co-cultures, neurons were plated on a rat astrocyte feeder layer as described previously [40]. A suspension of LbL-based polyelectrolyte microcapsules was added into the culturing medium, typically in a volume of 50 μl, at different developmental stages: 7–30 days *in vitro* (DIV). The duration of incubation varied – typically from 5 to 24 h – and in some experiments, for several days after capsule administration. After incubation, cell cultures were washed out with a medium to remove non-adherent microcapsules.

### 2.6 Two-photon excitation (2 PE) imaging in live cells

For live-cell 2 PE imaging, neurons were labelled with one of the lipophilic dyes, cyanine dye DIOC16(3) or carbocyanine dye DID (Biotium, UK), to provide a stable labelling of the lipid bilayer membranes. In some cases, a lipophilic dye, Nile Red, was used for the membrane staining. For membrane staining, cultured cells were incubated with a dye at a concentration of 6–10 μM for 30–45 min. After washing out the dye, a coverslip with cultured cells was transferred into an experimental chamber mounted on a stage of a Radiance 2000 imaging system (Zeiss-Bio-Rad) or a Femtonics imaging system optically linked to a Ti: Sapphire Mai-Tai femtosecond pulse laser (Spectraphysics-Newport). For live-cell imaging, recordings were performed in an artificial cerebrospinal fluid (aCSF) containing (in mM) 126 NaCl, 3 KCl, 2 MgSO_4_, 2 CaCl_2_, 26 NaHCO_3_, 1.25 NaH_2_PO_4_, 10 D-glucose saturated with 95 % O_2_ and 5 % CO_2_ (pH 7.4; 300–310 mOsmol) at 31–33 °C. The laser power was always kept below 6–8 mW during imaging to minimise phototoxic damage to the cells during scanning. Various digital zooms were used to visualise the microcapsule-conjugated fluorescence (Fig. 1D) relative to the two peaks of cell membrane fluorescence (lipophilic dyes; Fig. 1E). Appropriate optical filter sets were used to split the fluorescent signals between two emission channels. Z-stacks were collected using high digital magnifications and a frame mode of 512×512 pixels (typically 20–40 optical sections in 0.5–1.0-μm focal steps).

### 2.7 Electrophysiology

Electrophysiological recordings were carried out in whole-cell configuration to confirm the functional activity of neurons with microcapsules intracellularly, as in our previous studies [29,41,42]. For the recordings, neuronal cultures were transferred to a recording chamber mounted on an upright Olympus microscope and were perfused with an aCSF of the same composition as above (see 2.6). The recording electrodes had resistance of 2.5–4.5 MΩ when filled with the intracellular solution containing (in mM) 126 K-gluconate, 10 HEPES, 4 KCl, 4 MgCl_2_, 2 BAPTA, 4 Mg-ATP and 0.4 GTP-Na (pH 7.2 with KOH, osmolarity ∼290 mOsmol). Recordings were made using a Multipatch 700B amplifier controlled by the pClamp10.2 software package (Molecular Devices, CA, USA). Once the whole-cell configuration was established, neurons were monitored for changes in their intrinsic membrane properties. To elicit action potential (AP) firing, the series of sub- and supra-threshold rectangular current pulses (500–1000 ms duration) were applied with a gradually increased stimulus intensity. Recordings of neuronal firing discharge were collected at different times for at least half an hour of testing individual neurons.

### 2.8 Live-cell tracking of macropinosomes and lysosomes

To investigate the cellular mechanisms involved in capsule internalisation, cultured hippocampal neurons were incubated with microcapsules in the presence of labelled dextran, a biologically inert hydrophilic polysaccharide with a high molecular weight (MW). Specifically, we used Texas Red™ dextran of 3000 MW, neutral, which we added to the cells for 5–8 h before starting imaging cells. Prior to imaging, the cells were washed to eliminate any background fluorescence from extracellularly remaining dextran.

For lysosomal labelling, we used the highly selective Cell Light Lysosomes-GFP (Thermo Fisher Scientific), which is a BacMac 2.0 expression vector that encodes a fusion of GFP with the targeting sequence from lysosomal-associated membrane protein 1 (Lamp1). To express this vector, we transduced hippocampal neurons at 14 DIV by adding the vector at a ratio of 30 particles per cell, following the manufacturer's guidelines. Effective transduction was typically achieved after 16 h of incubation or longer. Lysosomes were then monitored using 2 PE imaging in live neurons.

### 2.9 Multiplexed 2 PE imaging *in vivo*

All animal procedures were conducted in accordance with the European Commission Directive (86/609/EEC) and the United Kingdom Home Office (Scientific Procedures) Act (1986) with project approval from the Institutional Animal Care and Use Committees of the University College London. All animals were maintained in controlled environments as mandated by national guidelines, on 12-h light/dark cycles, with food and water provided *ab libitum*.

#### 2.9.1 Cerebral delivery of microcapsules

Wild-type C57BL/6J mice (Charles River Laboratories) were used at the age of 1.5–2-month-old. A suspension of microcapsules was delivered into the somatosensory cortex during aseptic surgical procedures, as described in detail previously [43,44]. In brief, perioperative multimodal analgesia was carried out with buprenorphine (60 μg kg^−1^, s.c.) and lidocaine (2.5 %) topically applied to the surgical site. Isoflurane was used for anaesthesia throughout the surgical procedure: 4–5% v/v for induction and 1.5–2.5 % v/v for maintenance, controlled with pedal reflex. Body temperature was maintained at ∼37.0 °C using a feedback rectal thermometer and heating blanket. After localising bregma in the mouse head fixed in a stereotaxic frame, a local opening through the scull (∼1 mm diameter) was made with a high-speed dental drill at the coordinates for the somatosensory cortex (relative to bregma: AP: −1.5 mm, ML: −3 mm). Microcapsules were injected using a Hamilton syringe stereotactically guided to a depth of 0.5 mm beneath the cortical surface, under the control of a microinjection pump at a low rate in a total volume of 200–250 nl. Once delivery was completed, a needle was left in place for 5–10 min before being retracted. The surgical wound was closed, Metacam (1 mg kg^−1^, s.c.) and saline (0.5 ml) were administered, and the animal was placed in a heated chamber and observed until a full recovery.

#### 2.9.2 Surgical procedures: head plate installation, craniotomy, durotomy

Mice were prepared for craniotomy as described above and previously [[43], [44], [45]]. Once the animal was secured and deeply anaesthetised, the skull's right frontal and parietal bones were exposed; the area was cleaned and coated with tissue adhesive (3M Vetbond) to facilitate head plate installation. A custom-made head plate was affixed over the right somatosensory cortex (the targeted injection site) and secured with dental cement (Superbond, Sun Medical Co. Ltd., Japan). Once the headplate was fixed and the cement components cured, the animal was secured in a custom-built head fixation frame. A craniotomy of ∼3 mm diameter was performed over the somatosensory region using a high-speed hand drill. After sufficiently thinning the skull and superfusing its surface with saline, the skull flap was removed using fine-tipped forceps. Immediately after opening, the brain was superfused with sterile saline. Durotomy was carried out using 28G needles with hand-made curved tips, avoiding penetrating or damaging the pia mater. After completing the surgery, the anaesthesia regime was switched from inhalation to i.p. injection, using a mixture of fentanyl (0.03 mg kg^−1^), midazolam (3 mg kg^−1^), and medetomidine (0.3 mg kg^−1^), for the subsequent imaging in the anaesthetised animal.

#### 2.9.3 Multiplexed 2 PE imaging *in vivo*

A lipophilic dye DiO was applied onto the surface of the exposed cortex at a volume of 100 μl for 60–90 min, taking care to cover the open brain area fully. After loading the dye, the animal was transferred to the imaging system (Femtonics, Budapest) linked to the tuneable femtosecond pulse laser MaiTai (SpectraPhysics-Newport) for multiplex imaging. The anaesthetised animal was secured on a custom-built stage via the installed head plate under an XLPlan N25×/1.05 NA water immersion objective coupled to a green lamp illumination. 2 PE acquisitions were performed with laser at 820 nm to visualize areas with microcapsules (RITC signal, red channel) and cell membrane staining (DiO signal, green channel). Imaging was performed within superficial cortical layers, up to 200–250 μm depths. Imaging settings were adjusted to provide optimal recording conditions, adjusting laser intensity to minimise photobleaching. Various digital zooms were used to overview the brain regions containing microcapsules and to scan selected areas for visualising microcapsules relative to individual cells. After selecting the areas, 2 PE images were acquired as Z-stacks using Galvo frame scan mode.

### 2.10 Image analysis

High-resolution images acquired as Z-stacks were analysed off-line to plot profiles for each of the fluorescent signals: one – conjugated to the microcapsule shell (FITC or TRITC) and another – to the plasma membrane of individual cells (DID, DiO or Nile Red). Z-profiles of cell membrane signal were plotted against microcapsule fluorescence to assess whether microcapsules are present intracellularly. Microcapsules having their fluorescent signal within the two-peak fluorescent profile of the cell membrane were considered intracellularly internalised. The ImageJ software (NIH, Bethesda, USA) was used for the analyses.

### 2.11 Statistical analysis

All data are presented as mean ± standard error of the mean, unless specified otherwise, with the sample size *n* typically referring to the number of cells analysed or microcapsules as specified. A Student's *t*-test (two-tailed paired or unpaired) was used where appropriate to determine statistical differences between the experimental groups. A Fisher's exact test was used to assess whether variables in the proportions of datasets are random. A *p-*value of less than 0.05 was considered as a rejection level for the null hypothesis "no mean difference".

## 3 Results

### 3.1 Microcapsule uptake by differentiating N2A cells of neuronal phenotype

The intracellular uptake of nanoparticles fabricated using various techniques and material compositions has been well-documented in cell lines, including those of a neuronal phenotype, such as differentiated PC-12 cells [26], SH-SY5Y cells [46,47], and human cortical neuron cell line [48]. However, the efficacy of cellular uptake of microcarriers (micron size) remains unclear, with the only attempt reported for synthetic polymeric microcapsules internalised by B50 cells of a brain tumour line [49]. We therefore sought to examine the ability of neurons to internalise microcapsules, firstly using cell lines of neuronal phenotype, the differentiating N2A cells.

Synthetic PAH/PSS or biodegradable PArg/DS microcapsules were engineered with a hollow core structure (Fig. 1A). The measured zeta-potential of either sample was −18.2 ± 2.8 mV and −16.3 ± 4.0 mV for PAH/PSS and PArg/DS microcapsules, respectively. SEM images of the dispersed suspension of the fabricated microcapsules confirmed the capsule shell collapsing upon drying, indicating the dissolution of the calcium carbonate cores for the PAH/PSS- (Fig. 1B, left image) and PArg/DS-based microcapsules (Fig. 1C, left image). The microcapsules had an average diameter of 2–3 μm for either type: 1.9 ± 0.4 μm (n = 260) for the PAH/PSS (Figs. 1B) and 2.75 ± 0.59 μm (n = 301) for PArg/DS microcapsules (Fig. 1C). Two-photon excitation (2 PE) microscopy confirmed the microcapsule size and displayed a stable fluorescent signal mediated by FITC (Fig. 1D) or TRITC immobilised within the shell (Suppl. Fig. 1). A suspension of microcapsules was added to differentiating N2A cells, and high-resolution 2 PE imaging was performed in live cells at different time points of incubation after labelling cell lipid bilayer membranes with a lipophilic dye followed with 3D reconstruction for the cell membrane-mediated fluorescence (Fig. 1E) and microcapsule-conjugated fluorescence (Fig. 1D) using various digital zooms for collecting Z-stacks (see Methods for detail).

**Fig. 2:**
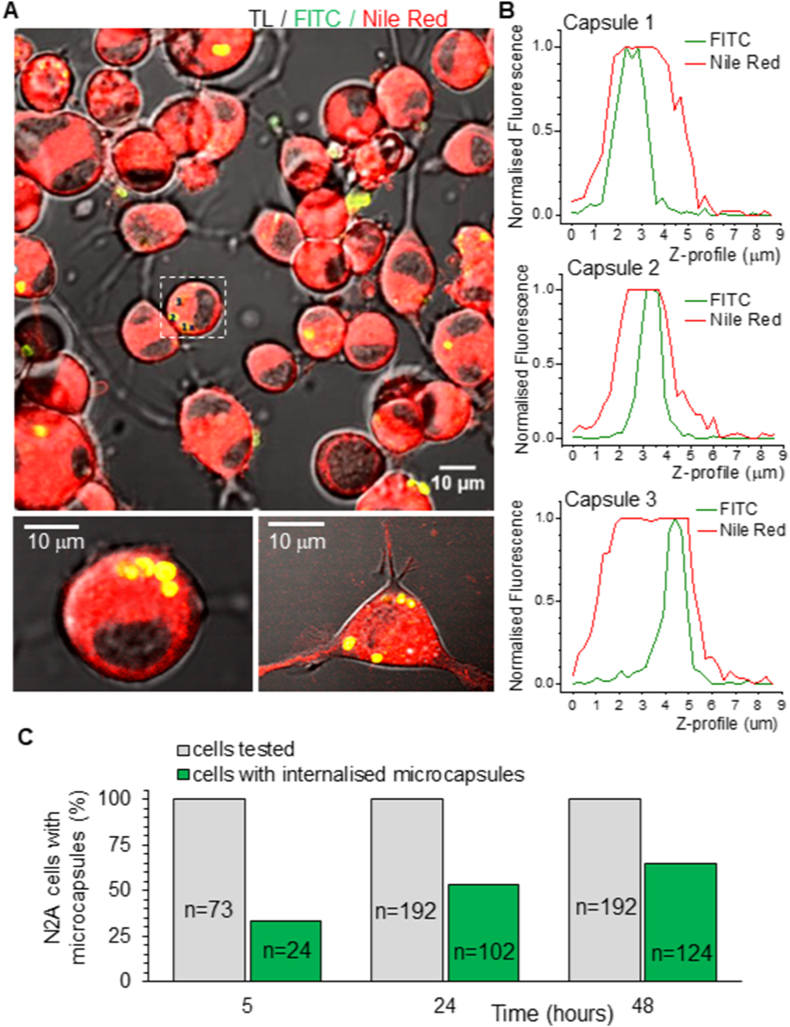
N2A cells of neuronal phenotype actively uptake microcapsules. (A) Merged 2 PE image of differentiating N2A cells in three channels (transmitted light, TL, green, FITC labelling PAH/PSS microcapsules, and Nile Red for cell membrane staining, red channel). Lower images are high-resolution 2 PE images of individual cells with a few microcapsules internalised. (B) Analysis of fluorescence signal profiles for individual PAH/PSS microcapsules (FITC, green channel) and the cell plasma membrane (Nile Red, red channel), demonstrating three microcapsules internalised by one cell (shown in the image by dotted square). (C) Statistical summary of the time-dependent internalisation of polyelectrolyte PAH/PSS microcapsules by differentiating N2A cells. Data are shown as mean; the numbers of cells scanned are indicated.

We found that differentiating N2A cells exhibit a strong capacity to internalise polymeric microcapsules, whether of PAH/PSS composition (Fig. 2A) or PArg/DS composition (Suppl. Fig. 2). Our experimental design ensured accurate visualisation of the microcapsule localisation within the cell by employing 3D reconstruction of the cell membrane-bound fluorescence (Nile Red signal, red channel) and microcapsule shell fluorescence (FITC signal, green channel) for each tested cell. Signal profile analyses showed that capsule-associated fluorescence was localised within the cell membrane profile (Fig. 2B), confirming the intracellular positioning of individual microcapsules. Furthermore, many cells internalised more than one microcapsule (Fig. 2A; Video 1 and 2). The plots in Fig. 2B demonstrate the profiles of three different microcapsules (2–3 μm size) internalised by the same cell (shown in Fig. 2A).

Supplementary data related to this article can be found online at https://doi.org/10.1016/j.mtbio.2025.101493

The following are the Supplementary data related to this article:

Video 1 Video 1

Video2 Video2

Having confirmed microcapsule internalisation, we next examined its time course. For these experiments, we used PAH/PSS-based microcapsules, chosen for their stable composition, which resists potential biodegradation and enables reliable time-dependent intracellular visualisation. Differentiating N2A cells were observed to internalise microcapsules within 5–6 h, with 33 % of all tested cells showing intracellular microcapsules within this timeframe (Fig. 2C). Concurrently, the majority of microcapsules appeared closely co-localised with and attached to the cell membrane, as confirmed by the matching fluorescent profiles of the microcapsule shell and the plasma membrane. The internalisation rate increased with time. At day 1 of incubation with microcapsules, 53 % of cells had internalised microcapsules (*p* = 0.0038, n = 102; two-tailed Fisher's exact test compared with 5 h). The proportion of cells with internalised microcapsules reached 65 % on day 2 (*p* = 0.0293, n = 124; two-tailed Fisher's exact test compared with day 1 and *p* < 0.0001 compared with 5 h). Notably, after two days of incubation, we observed many cells with microcapsules visualised inside the cell nucleus. These data confirm that cultured cells of neuronal phenotype uptake 2–3 μm polyelectrolyte capsules in a time-dependent manner. Also, it indicates the high biocompatibility of the fabricated microcapsules that were present either extracellularly or intracellularly.

When investigating the time-dependent internalisation of biodegradable PArg/DS-based microcapsules, we found that differentiating N2A cells internalised these microcapsules within 4–6 h, similar to the results observed with PAH/PSS-based microcapsules (Suppl. Fig. 2). The number of intracellular microcapsules increased with incubation time, with cells containing 1 to 5 microcapsules after 24 h. However, between 24 and 48 h, we observed a significant change in the shape of PArg/DS-based microcapsules inside the cells, transitioning from spherical to flattened shapes (Suppl. Fig. 3). This alteration suggests ongoing biodegradation of the intracellular microcapsules, consistent with our previous studies that tracked similar microcapsules until their complete degradation *in vivo* [29]. In contrast, microcapsules remaining outside the cells showed no changes in shape or fluorescence intensity, even after 72 h in the culture medium (Suppl. Fig. 3). These findings suggest that the intracellular biodegradation of PArg/DS-based microcapsules in the present settings occurs more rapidly than in the tissue (extracellular environment), resembling the behaviour of biodegradable polymeric capsules observed in other cell types [37,50].

### 3.2 Microcapsule uptake by cortical and hippocampal neurons *in vitro*

Given that differentiating N2A cells are highly sensitive to toxic environments and are commonly used for cytotoxicity testing [38,51], our data demonstrate that the fabricated microcapsules, whether PAH/PSS- or PArg/DS-based, exhibit high biocompatibility. This observation supports the microcapsule suitability for subsequent testing in primary brain neurons.

**Fig. 3:**
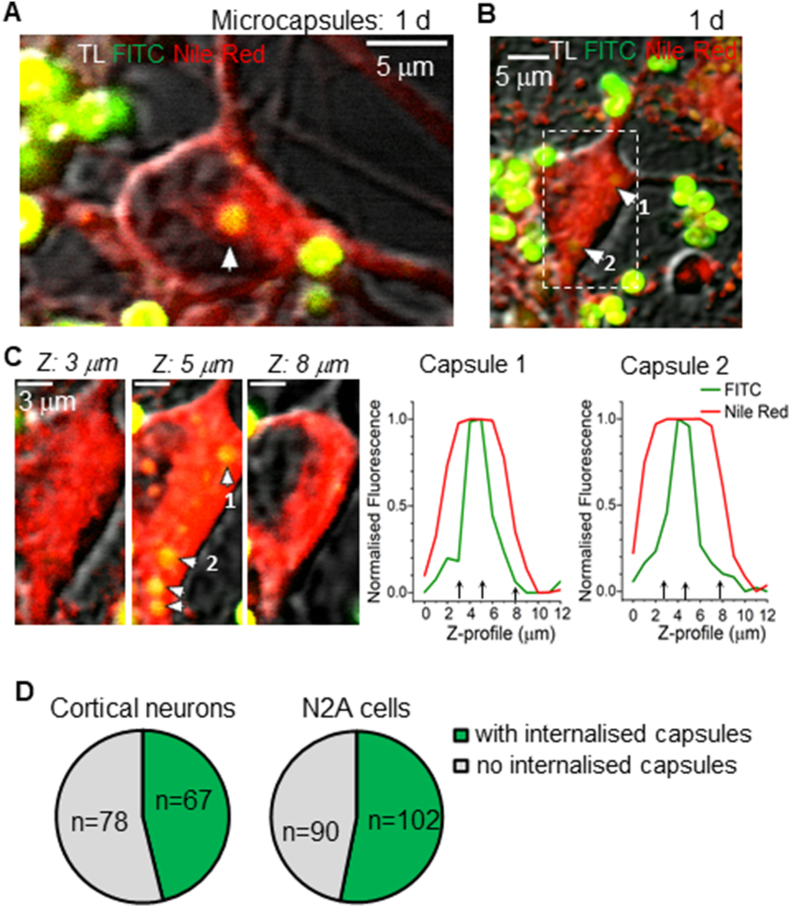
Microcapsule internalisation by cortical neurons *in vitro*. (A–B) Live-cell high-resolution 2 PE images of cultured cortical neurons supplemented with a suspension of PAH/PSS microcapsules, showing intracellular microcapsules (depicted by arrows). Images are merged transmitted light channel (TL), green (FITC) and red fluorescent channels (Nile Red). (C) Images of a cortical neuron (shown in B by a dotted rectangle) at different focal planes and Z-reconstruction of the fluorescence signal profiles (right plots) for FITC labelling microcapsule (green) and Nile Red staining neuronal membrane (red). (D) Statistical summary of the proportion of cortical neurons and differentiating N2A cells containing intracellular particles after 1 day of incubation with PAH/PSS microcapsules*.* The number of cells scanned is indicated.

To investigate whether brain neurons can internalise polyelectrolyte microcapsules, we added a suspension of PAH/PSS- or PArg/DS-based microcapsules to primary cortical or hippocampal neuron cultures and performed high-resolution live-cell imaging at various time points post-incubation. Fig. 3A and B show cortical neurons (7–8 DIV) after non-attached PAH/PSS-based microcapsules were washed away, revealing that the remaining microcapsules were densely attached to membrane structures, either on the cell bodies (somata) or neurites. To confirm microcapsule internalisation, we employed the same experimental design as described above, plotting the FITC fluorescence of the microcapsule shell (green channel) against the Nile Red-stained cell membrane (red channel) for individual cells. Z-profile analyses (∼0.5 μm resolution) verified the intracellular localisation of PAH/PSS-based microcapsules within cortical neurons. Notably, some neurons contained two or more internalised microcapsules after 1 day of incubation (Fig. 3B and C). Out of all the cortical neurons scanned at high resolution (n = 145), approximately 46 % contained microcapsules intracellularly (Fig. 3D). The proportion of cortical neurons with internalised microcapsules at day 1 of incubation was similar to that in the differentiating N2A cells at day 1 (*p* = 0.23, two-tailed Fisher's exact test for cortical neurons compared with N2A cells).

**Fig. 4:**
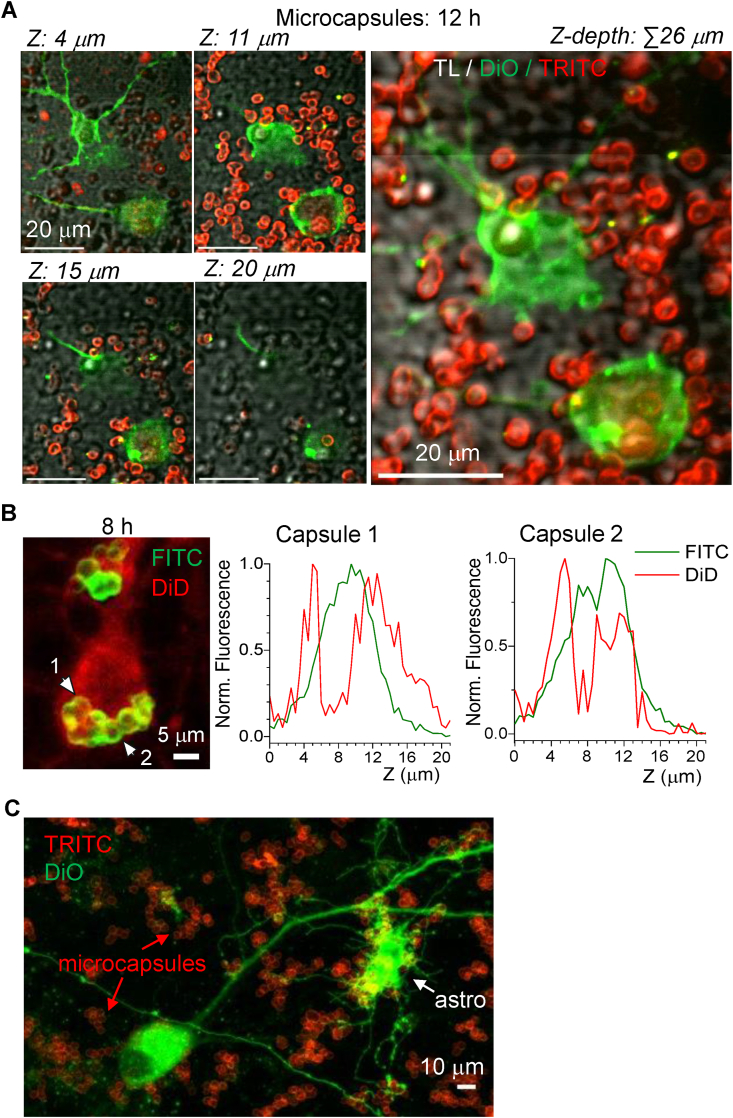
Microcapsule uptake by hippocampal neurons *in vitro*. (A) 2 PE images of two hippocampal neurons taken across different focal planes (Z-depth) as noted, with Z-projection on the right after 12 h of incubation with PArg/DS-based microcapsules. Images are merged red channel (TRITC, microcapsule fluorescence), green (DiO, neuronal plasma membrane) and transmitted light (TL) channels; λ_x_^2PE^ = 910 nm. (B) Image of a hippocampal neuron at high resolution showing at least two microcapsules internalised (1 and 2) after 8 h of incubation with PArg/DS-based microcapsules. Plots show Z-profiles of FITC signal (microcapsule shell) relative to DiD fluorescence (neuronal plasma membrane) for capsules 1 and 2 indicated on the left image. (C) Image of hippocampal neuronal-astrocytic co-culture supplemented with PArg/DS-based microcapsules (8 h of incubation). Note astrocyte (‘astro’) with numerous internalised microcapsules.

In cultured hippocampal neurons (14 DIV), we tested the PArg/DS-based microcapsules, which displayed a similar profile as PAH/PSS-based microcapsules in cortical neuronal cultures. In particular, microcapsules were densely scattered around neuronal somata and along neurites (Fig. 4; Suppl. Fig. 4). Using 3D reconstruction for both plasma membrane and microcapsule shell fluorescence, we visualised the microcapsule-conjugated signal (FITC or TRITC) within the two-peak fluorescent profile for the plasma membrane (DiD or DiO staining), confirming that microcapsules are internalised inside neurons (Fig. 4A and B). In hippocampal neuronal-astrocytic co-cultures (14 DIV), microcapsules were co-localised with neurons and were notably clustered in astrocytes, distinguished by their characteristic morphology (Fig. 4C; Suppl. Fig. 5).

The uptake rate of PArg/DS microcapsules was relatively high in hippocampal neurons. We observed internalised microcapsules within 5–6 h post-application across independent culture preparations (n = 10 preparations). On day 1 of incubation with microcapsules, the proportion of hippocampal neurons with internalised microcapsules was ∼26 %, and it increased to ∼43 % after 2 days. Notably, microcapsules of the same composition and charge (−17.1 ± 3.7 mV) but of a larger size (5.1 ± 0.8 μm, n = 300; Suppl. Fig. 6A) were rarely observed inside neurons, while remaining adherent to neuronal structures (Suppl. Fig. 6C). However, high-resolution 2 PE scanning enabled us to confirm that the microcapsule shell co-localised with cell membranes even for particles of several microns in size. This suggests that the microcapsule fused with the membrane, allowing for potential delivery inside the cell (Suppl. Fig. 6B).

### 3.3 Mechanism of microcapsule internalisation; lysosomal escape

**Fig. 5:**
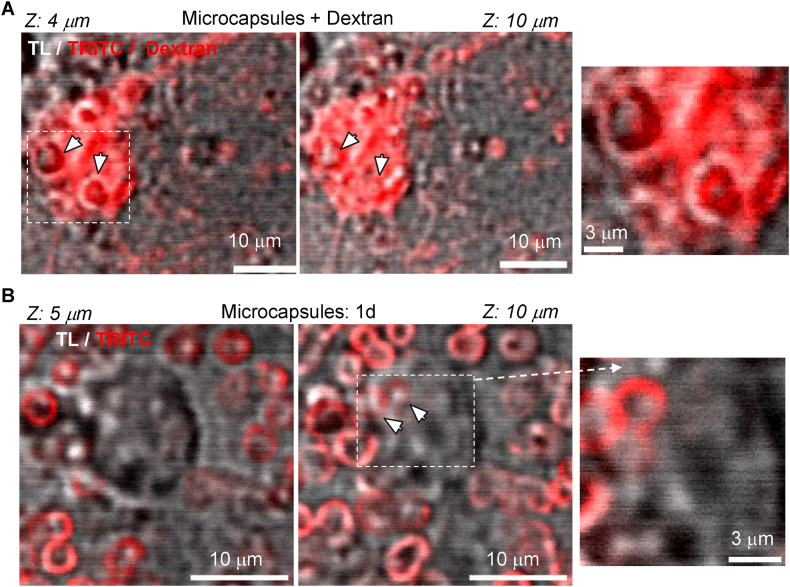
Neuronal uptake of microcapsules occurs via macropinocytosis. (A–B) 2 PE images of hippocampal neurons containing intracellular microcapsules after 8 h of incubation with PArg/DS-based microcapsules in the presence of Dextran Texas Red, 3000 MW (A) or without (B). Images show different focal planes across Z-depth, as noted. The right images show enlarged areas, indicated by the dotted squares in the left images. Images are merged red channel (TRITC, microcapsule fluorescence, and dextran) and transmitted light (TL) channel; λ_x_^2PE^ = 910 nm.

Various mechanisms can mediate the entry of engineered particles into cells, influencing the effectiveness of cargo delivery. Endocytosis plays a critical role across different cell types and serves as the primary pathway for the cellular uptake of nanoparticles, employing multiple endocytic mechanisms [52]. Given the micron-sized dimensions of the fabricated capsules, which effectively rule out the clathrin-dependent, clathrin-independent, or caveolae-mediated endocytosis pathways [53], we hypothesized that macropinocytosis plays a key role in the intracellular uptake of large volumes. To investigate this, we used fluorescent-conjugated high-molecular-weight dextran as a tracer to track intracellular fluorescence following microcapsule internalisation by brain neurons. Our experiments detected robust dextran fluorescence inside hippocampal neurons containing intracellular PArg/DS microcapsules (Fig. 5). Tracer fluorescence was observed in every imaged cell containing internalised microcapsules (n > 30), indicating that neurons can macropinocytose large volumes of material. These findings suggest that actin-driven macropinocytosis could be the primary mechanism underlying the uptake of microcapsules by neurons.

After their formation, macropinosomes interact with cellular compartments and undergo endosomal sorting. From early endosomes, cargo can be directed to late endosomes and subsequently to lysosomes, where hydrolytic and enzymatic digestion occurs, a common fate for nanoparticles [54]. Alternatively, the cargo can be recycled back to the cell surface or released into the cytoplasm. Our experiments with dextran revealed strong tracer staining throughout the entire neuronal cell body (Fig. 5A; Suppl. Fig. 7), suggesting that the tracer enters the cytoplasm following microcapsule uptake.

**Fig. 6:**
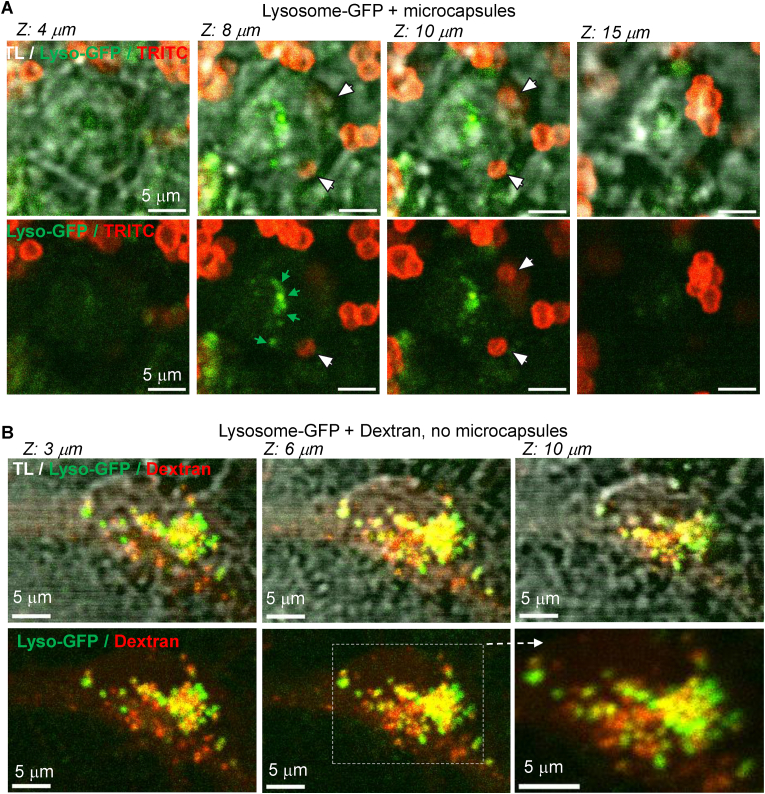
Internalised microcapsules escape lysosomal formation. (A) High-resolution 2 PE images of a hippocampal neuron expressing GFP BacMac 2.0, highly specific for lysosomal staining, at different focal planes across Z-depth, as noted, demonstrate no co-localisation between intracellular microcapsules (TRITC, red signal) and lysosomes (GFP, green signal). Upper raw, images are merged transmitted light (TL), green channel (GFP, lysosomes), and red channel (TRITC, microcapsule fluorescence); lower raw, same as above but with no TL channel; λ_x_^2PE^ = 910 nm. (B) High-resolution 2 PE images of a hippocampal neuron after GFP BacMac 2.0 transduction (>16 h) for labelling lysosomes after incubation with Dextran Texas Red, 3000 MW at different focal planes across Z-depth, as noted, demonstrate a high amount of intracellular macropinosomes (dextran, red signal) entrapped in lysosomes (GFP, green signal). Upper raw, images are merged transmitted light (TL), green channel (GFP, lysosomes), and red channel (Dextran Texas Red); lower raw, same as above but with no TL channel; λ_x_^2PE^ = 910 nm.

To investigate whether internalised microcapsules accumulate in lysosomes, we utilized the Lysosome-GFP BacMac 2.0 vector for specific labelling of lysosomal-associated membrane protein 1 (Lamp1). Following neuronal transduction, we visualised intracellular microcapsules and lysosomes using high-resolution 2 PE imaging in live hippocampal neurons. As shown in Fig. 6A, after more than 16 h of incubation with PArg/DS microcapsules, none of the detected intracellular microcapsules co-localised with lysosomes, which were distributed abundantly throughout the cytoplasm. Across Z-profile scans, we did not observe any GFP signal surrounding the microcapsules, further confirming the lack of co-localisation between intracellular microcapsules and lysosomes. To validate that macropinosomes are typically associated with lysosome formation, we performed additional experiments by incubating hippocampal neurons with fluorescently labelled dextran instead of microcapsules. These experiments revealed a substantial co-localisation of red and green vesicles within the same focal plane across multiple Z-depths (Fig. 6B), indicating a high level of co-localisation between the tracer and GFP-labelled lysosomes.

Together, these findings demonstrate that neurons internalise polyelectrolyte microcapsules via actin-driven macropinocytosis and that the microcapsules effectively evade lysosomal accumulation. This evasion is critical for accessing intracellular targets and achieving therapeutic effects post-uptake.

### 3.4 Microcapsules are biocompatible, with no changes to normal neuronal activity

In the next set of experiments, we employed the electrophysiological patch-clamp technique to visualise microcapsules within hippocampal neurons using whole-cell dialysis. After overnight incubation of neurons with a suspension of PArg/DS microcapsules, individual neurons were patched, allowing a bright morphological tracer Alexa Fluor to fill the intracellular space via the patch pipette. This was followed by high-resolution 3D scanning of the entire cell volume.

**Fig. 7:**
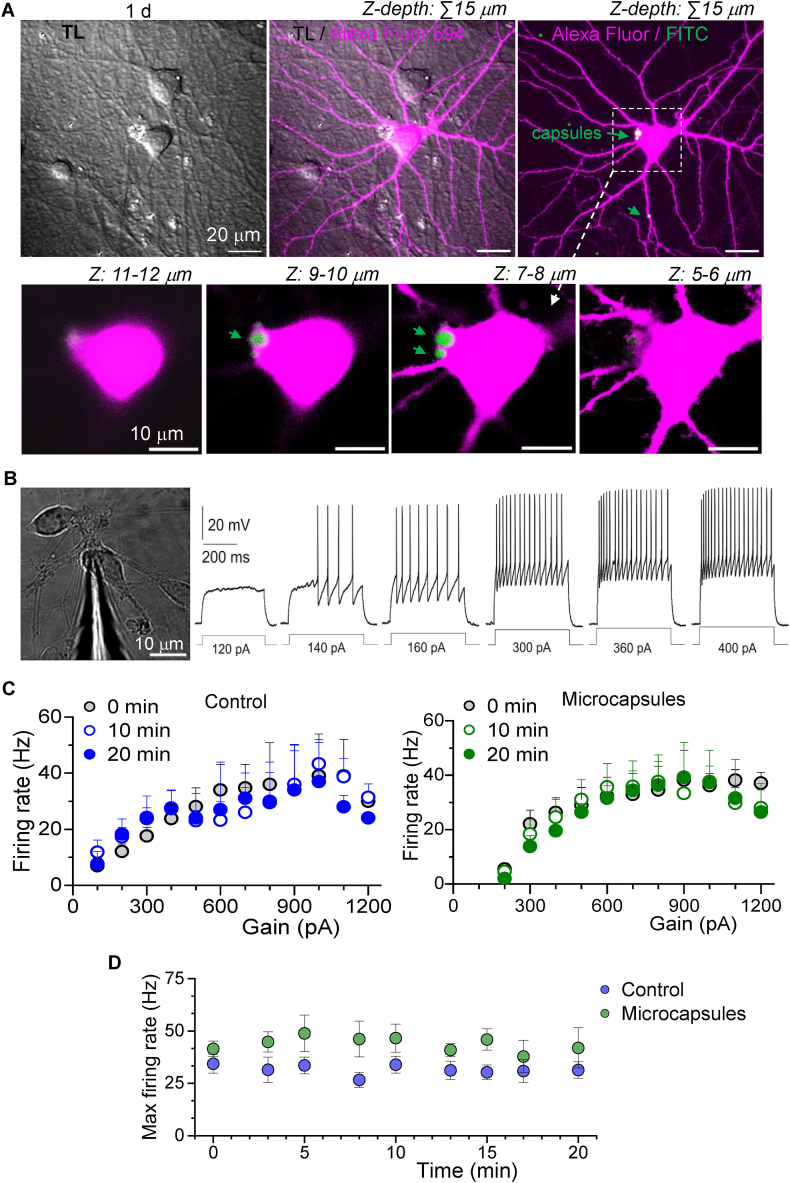
Visualisation of intracellular microcapsules with electrophysiological technique and monitoring firing activity of hippocampal neurons. (A) 2 PE images of hippocampal neurons in transmitted light (TL) channel (left image), red fluorescent channel (Alexa Fluor, right image), and merged red and green (FITC) channels (middle image), displaying a neuronal morphology with internalised microcapsules after 1 d of incubation with PArg/DS-based microcapsules *in vitro*. Lower panel, images of neuronal somata (dotted square on upper image) at different focal planes over Z-depth with two microcapsules inside the cell volume. (B) Snapshot of hippocampal neurons with a patch pipette in whole-cell configuration. Traces show original electrophysiological recordings of neuronal activity (action potential firing) recorded from the same neuron in response to depolarising stimulus of different intensities, starting from the subthreshold stimulus with gradually increased stimulus strength (shown on the bottom). (C) Statistical summary of the firing rate plotted against depolarising current stimulus analysed for each neuron tested (as shown on panel B) for the groups of control neurons (no microcapsules, n = 16) and neurons with microcapsules inside (n = 12) recorded over different time points of testing. Data are mean and s.e.m. (D) Statistical summary of the maximal firing rate analysed for control neurons (no microcapsules, n = 16) and neurons with intracellular microcapsules (n = 12), demonstrating similar electrophysiological activity between the groups of neurons in whole-cell recordings. Data are mean with s.e.m.

Fig. 7A shows hippocampal neurons at 14 DIV, with one neuron filled with Alexa Fluor (red channel), clearly outlining the cell morphology, including the somata, neurites, and dendritic spines. Three microcapsules (FITC, green channel) are visible, two inside the soma and another at a neurite branch. Z-scanning through the cell depth confirms that the somatic microcapsules are intracellular (Fig. 7A, lower images).

Using patch-clamp recordings, we next investigated whether intracellular microcapsules (without cargo) affected neuronal function. To do this, we monitored the electrical activity of hippocampal neurons over time by recording action potential (AP) discharges elicited by membrane depolarization in response to current stimuli of varying intensities, following the approach used in our previous studies [29,41,42]. Because the AP discharge is highly sensitive to changes in physiological conditions, it best reflects possible changes in neuronal excitability, representing a physiological readout of neuronal activity. Individual neurons were tested by first applying a subthreshold stimulus, followed by gradually increasing the stimulus intensity until the maximal AP discharge was elicited (Fig. 7B). We analysed the firing rate for each neuron and plotted it against stimulus strength, observing an exponential profile of neuronal excitability that plateaued at maximal levels. A statistical summary showed that these exponential profiles were comparable between the control group of hippocampal neurons without microcapsules (n = 16) and neurons with intracellular microcapsules (n = 12; Fig. 7C).

Additionally, we monitored neuronal excitability in real time, starting immediately after membrane breakthrough ("0″ time point) and at subsequent time intervals. A statistical summary revealed no changes in neuronal excitability over the course of the recordings in either the control group or neurons with intracellular microcapsules (Fig. 7C). Finally, we analysed the maximal firing rate for each tested neuron. Consistently, no significant difference was observed between the control group and neurons containing microcapsules (p > 0.05; Fig. 7D).

These findings indicate that polyelectrolyte-based microcapsules, once internalised, do not have detrimental effects on neuronal excitability or firing capacity. This aligns with our previous studies, which demonstrated that polyelectrolyte PArg/DS-based microcapsules (without payload) did not alter the biophysical properties of neuronal membranes, nor the parameters of individual AP spikes elicited by hippocampal neurons [29].

### 3.5 Microcapsules localise within the targeted brain region and undergo uptake by cortical neurons *in vivo*

**Fig. 8:**
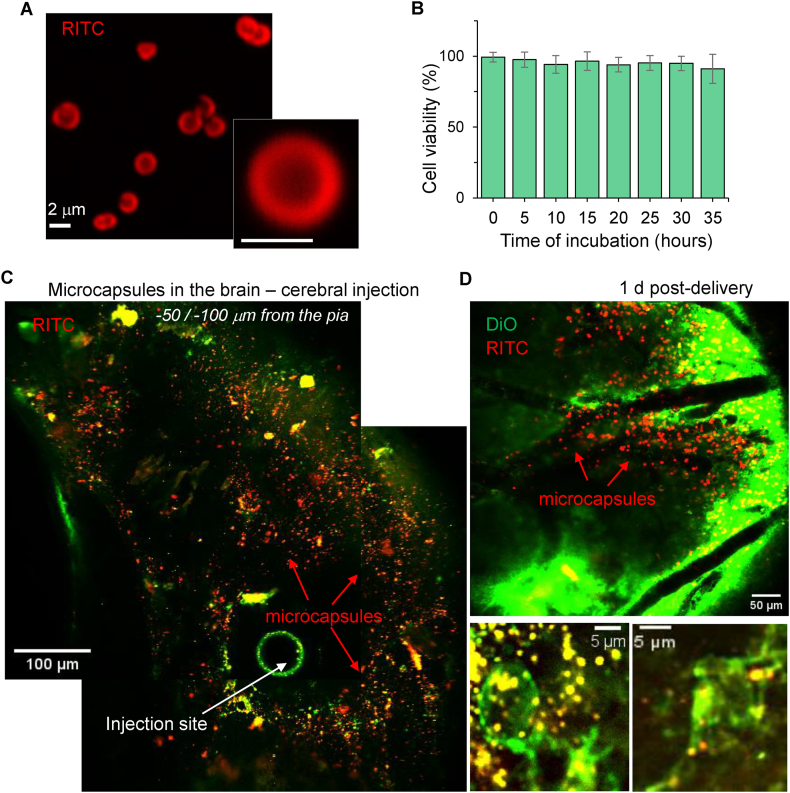
Localised microcapsule distribution within the targeted somatosensory cortex and microcapsule uptake by cortical neurons *in vivo.* (A–B) Image of a dispersed suspension of PAH/PSS-based microcapsules (RITC fluorescence) and cytotoxicity test (B) showing no changes in cell viability for differentiating N2A cells over time of incubation with microcapsules. Data are mean and s.d. Scale bar: 2 μm. (C–D) 2 PE images of microcapsule distribution (RITC fluorescence, red channel) within the somatosensory cortex 24–48 h after stereotaxic cerebral injection of PAH/PSS-based microcapsules *in vivo*. Note the injection site and localised microcapsule distribution within the targeted region (∼350–400 μm from the injection site) in C and D (top image). Lower images in D show individual neurons with intracellular microcapsules in the somatosensory barrel cortex. Images are merged red (RITC fluorescence) and green channels (lipophilic cyanine dye DIOC16, membrane staining). Images are average Z-profiles (3–5 focal planes); λ_x_^2PE^ = 820 nm.

Finally, we investigated the internalisation of microcapsules in the living brain. To enable the delivery of microcapsules directly into the brain parenchyma, we developed a stereotaxically guided intracerebral administration technique targeting the somatosensory cortex (see Methods). Notably, localised brain injections have been widely used in research to enable targeted drug delivery to specific brain regions [43,44,55] and also in clinics [56]. To ensure continued visualisation of microcapsules over longer time periods, we used the non-biodegradable PAH/PSS-based microcapsules, with RITC conjugated within their shell (Fig. 8A). Although our experiments above clearly demonstrated the high biocompatibility of these microcapsules, we validated it for the freshly made fabrications prior to injections *in vivo* (Fig. 8B).

A suspension of dispersed microcapsules was stereotaxically injected into the somatosensory cortex in a volume commonly used for intracerebral delivery (up to 250 nl). On day 2, we performed high-resolution 2 PE imaging *in vivo* to visualize the distribution of microcapsules within the targeted brain region. Our imaging revealed a successful dispersion of microcapsules within the somatosensory cortex, densely distributed approximately 350–400 μm from the injection site. Fig. 8C provides a stitched overview of the cortical region, showing the injection site and the localised presence of microcapsules within the area. The microcapsules were dispersed and scattered, ensuring precise delivery to the targeted region as intended. To visualise cells within the brain parenchyma, we used the lipophilic dye DiO for cell membrane staining. Using multiplexed high-resolution imaging with varying digital zooms, we observed numerous cortical neurons containing internalised microcapsules (Fig. 8D, lower images). Additionally, microcapsules were found to co-localise with neuronal processes.

**Fig. 9:**
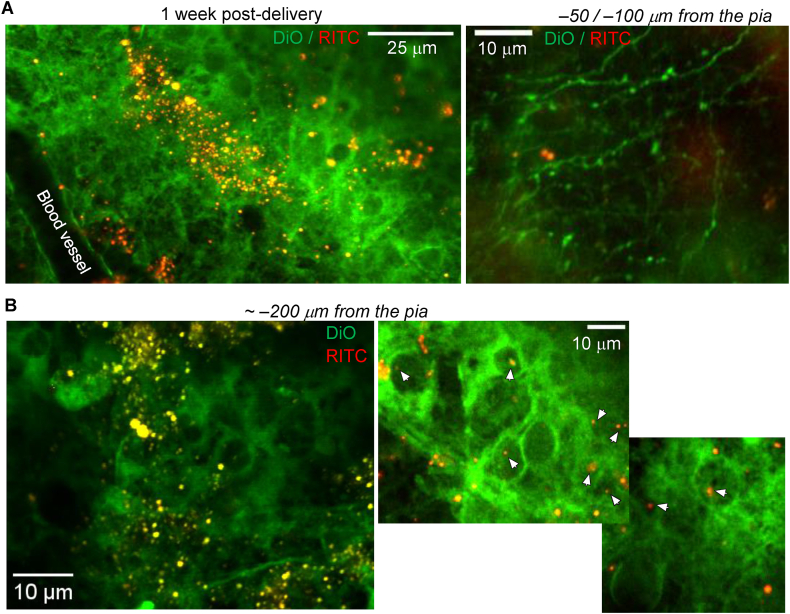
Long-term assessment of microcapsules in the somatosensory cortex *in vivo.* (A) 2 PE microscopy *in vivo* visualised PAH/PSS-based microcapsules (RITC fluorescence, red signal) across the targeted somatosensory cortex 5–7 days after stereotaxic cerebral injection. The left image shows an overview of microcapsule scatter; the right image notes sparsely distributed microcapsules across individual fibres. Images are merged red channel (RITC, microcapsule shell) and green channel (lipophilic cyanine dye DIOC16, cell membrane staining). (B) Images of the somatosensory barrel cortex (approximately −200 μm from the pia), showing individual neurons with intracellular microcapsules. Images are merged red (RITC fluorescence) and green channels (DIOC16, membrane staining); λ_x_^2PE^ = 820 nm.

Finally, we validated the biocompatibility and safety of microcapsules delivered to the brain by monitoring animals for up to a week following the *in vivo* delivery of microcapsules into the somatosensory cortex. Animals were closely observed for any health concerns potentially associated with the persistent presence of non-degradable PAH/PSS-based microcapsules. The animals displayed no health issues and behaved normally, comparable to their littermates that did not receive microcapsule administration. These observations indicate that the microcapsules do not induce adverse effects while present in the brain, consistent with our *in vitro* findings. Fig. 9 illustrates the microcapsules within the targeted brain region at various depths. We observed microcapsules distributed along neuronal fibres (Fig. 9A right) and across neuronal soma in somatosensory layers I–II. Numerous cells containing microcapsules were clearly visualised within the cell body volume (Fig. 9B). Altogether, these results confirm the neuronal uptake of polymeric microcapsules in the intact brain and validate their long-term biocompatibility, whether present extracellularly or intracellularly.

## 4 Discussion

Drug delivery to the brain remains a significant challenge, often accompanied by adverse effects resulting from high drug concentrations. Nanoengineered microencapsulation offers a promising solution, enabling precise targeting while delivering low-volume, high-concentration drugs to brain cells, followed by the controlled release of encapsulated compounds, as demonstrated in previous studies [29,30,49]. Intracellular delivery of bioactive compounds can provide controlled targeting and help overcome challenges related to the bioavailability and pharmacokinetics of compounds that act intracellularly but typically require external application at high concentrations to reach their targets. While the internalisation of ligand-targeted nanoparticles for intracellular drug delivery has been extensively studied across various tissue types (for review, see Ref. [57]), our understanding of their efficacy in neurons, particularly in the living brain, remains limited. Only a few studies have investigated the cellular uptake of nanoparticles in neurons. For example, lipid-based nanocarriers have been shown to deliver genetic materials to primary rat cortical neurons and a human cortical cell line [48], and magneto-liposomes have been internalised by human and rat astrocytes as well as endothelial cells [58]. Here, we present a proof-of-concept for intracellular delivery to brain neurons using synthetic polyelectrolyte-based microcarriers, specifically polymeric microcapsules fabricated via the LbL-technique.

Our high-resolution live-cell multiplex imaging demonstrates, for the first time, that brain neurons can internalise microcapsules made of polyelectrolyte polymers with dimensions of a few microns. We confirmed the microcapsule uptake in N2A cells of neuronal phenotype (Fig. 2), hippocampal neurons (Fig. 4, Fig. 6, Fig. 7), cortical neurons *in vitro* (Fig. 3), and cortical neurons *in vivo* (Fig. 8, Fig. 9). In earlier studies, microcapsules of similar fabrication were also found to be internalised by a tumour cell line (B50 cells) following overnight incubation [49]. Our data, however, indicate that internalisation can occur within just a few hours. Furthermore, brain cells can internalise more than one microcapsule, while cells of a neuronal phenotype (cell lines) can take up several particles. A comparable time window and high internalisation rate have also been observed for nano-vehicles [58].

The time-dependent internalisation of particles correlates with their size, a key parameter that predominantly influences the uptake pathway [59]. Larger particles are internalised less efficiently, as demonstrated by our results showing reduced uptake of microcapsules larger than 4–5 μm by brain neurons (Suppl. Fig. 6). Additionally, our previous studies indicated that the size of microcapsules is a critical factor influencing their circulation time in the bloodstream *in vivo* [60]. Specifically, 30 % of 1-μm magnetic Parg/DS microcapsules remained circulating for over 3 min after injection, whereas microcapsules measuring 2.7 μm and 5.5 μm were not detected at the same time point. Microcapsule fabrications often exhibit a degree of polydispersity. In this context, a suspension of particles with varying sizes can facilitate cellular uptake in a relatively controlled manner [61]. Our typical fabrications included microcapsules, ranging from 1 to 3 μm (Fig. 1). Although polydispersity can influence the loading efficiency of drugs into the capsules, this issue may be mitigated by the tendency of cells to internalise smaller capsules preferentially. This behaviour could ultimately result in a more uniform distribution of the released drug across targeted regions or cell populations. In our previous studies, we used electrophysiology to monitor drug release from PArg/DS microcapsules, demonstrating that the encapsulated sodium channel blocker gradually suppressed neuronal excitability regardless of the number of particles delivered intracellularly via patch pipette [29]. In addition to size, particle composition also significantly influences cell membrane interactions. For examples, incorporating positively charged poly-L-arginine (PArg) can enhance electrostatic interactions with the plasma membrane, potentially facilitating cellular uptake. Our recent studies demonstrated that microcapsules with a higher positive charge, achieved by including positively charged polyelectrolytes in the outer layers, are more effectively captured by cells [62]. However, these microcapsules also exhibit increased cytotoxicity. Positively charged microcapsules are more prone to aggregation, which becomes a critical obstacle for *in vivo* applications.

Interestingly, intracellular incorporation of microcapsules can be achieved with virtually any polymeric capsule of a size suitable for intracellular uptake, utilising techniques such as electroporation and mechanical forces [63]. The characteristics of microcapsule deformation during electroporation-driven intracellular incorporation play a critical role in defining the drug release profile, as they are influenced by the degree of polymer deformation. In contrast, the intracellular uptake observed in neurons during our study occurred spontaneously, without applying external stimuli or forces. This uptake was primarily mediated by a micropinocytosis mechanism, which enables neurons to internalise large materials [52]. Consequently, we did not observe any significant changes in intracellular microcapsules shortly after uptake. However, biodegradable polymeric materials became compressed over time, with intracellular microcapsules appearing flattened and shrunken after more than 1 day. This suggests their degradation within the more acidic intracellular environment, rich in endogenous enzymes. Additionally, intracellular microcapsules are subjected to osmotic and cellular mechanical forces, which correlate with the number of polymer layers and, consequently, the mechanical properties of the fabricated capsules [64].

Our study explored the use of microcarriers in the intact brain, an area that has not been previously investigated. Utilising multiplexed 2 PE microscopy *in vivo*, we visualised the distribution of microcapsules following intracerebral administration to the targeted brain region (the somatosensory barrel cortex). We demonstrated the uptake of polymeric microcapsules by cortical neurons and their co-localisation with individual neuronal fibres. The integrity of the blood-brain barrier (BBB) is well-known to prevent the penetration of large compounds into the brain parenchyma. While previous studies have reported that nanoparticles or nanoliposomes can cross the blood-brain barrier (BBB), the ability of larger microcarriers to do so remains unexplored. Fluorescent signal readouts from these studies indicated that nanocarriers were primarily distributed in the liver after intravenous administration, with minimal presence in the brain [65,66]. To address the challenges of compromised carrier delivery through the BBB [67], we developed a method of direct intracerebral administration of a dispersed suspension of microcapsules to specific brain regions via microinjection. This approach enabled direct access to the brain parenchyma in a healthy brain environment. Future studies could explore alternative delivery methods, such as infusion into the cerebrospinal fluid or systemic delivery via blood flow, a common clinical approach for brain access. This strategy may be particularly viable for certain neurological disorders where BBB breakdown occurs (for review, see Ref. [68]).

Despite their proven effectiveness in carrying payloads, microcapsules must meet stringent standards for biological safety and low cytotoxicity. Our previous studies have demonstrated the biocompatibility and safe use of LbL-fabricated polyelectrolyte microcapsules in primary neurons [29,30] and peripheral tissues *in vivo* [29]. Additionally, PArg/DS-based microcapsules exhibit progressive biodegradation after *in vivo* injection, with no detectable microcapsule-conjugated fluorescence observed in peripheral tissue samples between 1 and 5 weeks post-injection. In the present study, we further validated the biosafety of microcapsules in brain neurons by assessing neuronal excitability and firing activity in hippocampal neurons, a neuronal subtype highly susceptible to detrimental environments. Hippocampal neurons containing polymeric microcapsules showed no changes in firing discharge – a key measure of neuronal excitability – over the testing period (Fig. 7). These findings confirm the safety of polymeric microcapsules and support their potential therapeutic applicability in neurology.

## 5 Conclusions

Our study provides compelling evidence that brain neurons can internalise polyelectrolyte-based polymeric microcarriers composed of either stable or biodegradable materials. We confirm that the internalisation of micron-sized capsules is time-dependent and occurs via actin-driven macropinocytosis, enabling capsules to enter the cytoplasm without becoming trapped in lysosomes. Importantly, the presence of polyelectrolyte-based microcapsules, whether extracellularly or intracellularly, did not affect neuronal firing activity or key aspects of animal behaviour, even during prolonged presence in the mouse brain.

The demonstrated ability of LbL-fabricated polyelectrolyte-based polymeric microcapsules to penetrate membrane-bilayer barriers in brain neurons, combined with their increased cargo-carrying capacity, tuneable drug-release pharmacokinetics, and biocompatibility, underscores their significant potential as a drug delivery platform for neurology. These microcapsules offer promising opportunities for precision targeting of brain neurons, paving the way for targeted drug delivery and precision treatment of various brain disorders.

Further research is needed to explore the applications of polymeric microcapsules in neuropathological conditions, particularly in the context of targeted drug delivery to damaged brain regions in a range of CNS pathologies.

## CRediT authorship contribution statement

**Olga Kopach:** Writing – original draft, Visualization, Validation, Methodology, Investigation, Funding acquisition, Formal analysis, Data curation, Conceptualization. **Olga A. Sindeeva:** Visualization, Validation, Methodology, Investigation, Formal analysis. **Kaiyu Zheng:** Visualization, Software, Methodology. **Eleanor McGowan:** Methodology. **Gleb B. Sukhorukov:** Methodology, Funding acquisition, Conceptualization. **Dmitri A. Rusakov:** Supervision, Resources, Funding acquisition, Conceptualization.

## Declaration of Competing Interest

The authors declare that they have no known competing financial interests or personal relationships that could have appeared to influence the work reported in this paper.

## Data Availability

Data will be made available on request.

## Appendix A Supplementary data

Fig. S1 A suspension of the Parg/DS-composed microcapsules before administration to neuronal cultures *in vitro*. The image is taken with two-photon excitation (2 PE) microscopy at λ_x_^2PE^ = 960 nm. Fig. S1

Fig. S2 Differentiating N2A cells of neuronal phenotype effectively uptake polyelectrolyte PArg/DS-based microcapsules. Images show N2A cells after 4–5 h of incubation (top images) and 24 h of incubation with a dispersed suspension of microcapsules. Right images, enlarged cells (dotted square) with microcapsules inside. Scale bar: 10 μm. Merged red channel (TRITC, microcapsule shell) and transmitted light (TL) channel. Fig. S2

Fig. S3 Intracellular biodegradation of polyelectrolyte PArg/DS microcapsules by N2A cells of neuronal phenotype. Images of differentiated N2A cells after 24 h (left panel), 48 h (middle panel) and 72 h of incubation (right panel) with a suspension of PArg/DS microcapsules. The bottom row shows enlarged microcapsules (dotted square in images above) outside and inside the cells. Scale bar: 2 μm. The top row shows the merged red channel (TRITC, microcapsule shell) and transmitted light (TL) channel. Fig. S3

Fig. S4 Microcapsule uptake by hippocampal neurons *in vitro*. Two-photon excitation (2 PE) images of cultured hippocampal neurons (34 DIV) supplemented with a suspension of PArg/DS microcapsules across various focal planes as indicated. The lower image shows an enlarged snapshot of the indicated area (white dotted square in the top image). Images are merged transmitted light (TL), green fluorescent channel (DiO, plasma membrane staining) and red channel (TRITC, microcapsule shell). Fig. S4

Fig. S5 Microcapsule uptake by hippocampal neuronal-astrocytic co-cultures. Two-photon excitation (2 PE) images of hippocampal neuronal-astrocytic co-cultures supplemented with a suspension of microcapsules (8 h of incubation *in vitro*). The left image is the red fluorescent channel (TRITC fluorescence). Right image, the green channel (DiO, the plasma membrane staining). Fig. S5

Fig. S6 Neuronal uptake of larger-sized microcapsules. (A) SEM image of the PArg/DS-based microcapsules fabricated with a larger diameter (∼5 μm) (B) 2 PE image of a hippocampal neuron after 1 d incubation with larger diameter PArg/DS microcapsules (top) and Z-profiles (lower plots) for the microcapsule's shell (TRITC, red channel) and plasma membrane fluorescence (DiO, green channel) for two microcapsules (1 and 2) indicated on the upper image. (C) 2 PE image of a hippocampal neuron after 1 d incubation with large-sized PArg/DS microcapsules shows no intracellular microcapsules but co-localisation with neuronal structures. Fig. S6

Fig. S7 Microcapsule internalisation relates to macropinocytosis by hippocampal neurons. Two-photon excitation (2 PE) images of hippocampal neurons supplemented with a suspension of PArg/DS microcapsules in the presence of high molecular weight Dextran Texas Red (8 h of incubation *in vitro*). Images are merged transmitted light (TL) and red fluorescent channels (TRITC and dextran fluorescence) at different Z-depth, as noted. Fig. S7
