# Supplementary figures and images for: Brain neurons internalise polymeric micron-sized capsules: Insights from in vitro and in vivo studies
Source: Mater Today Bio. 2025 Jan 21;31:101493. doi: 10.1016/j.mtbio.2025.101493 (PMC11815287; doi:10.1016/j.mtbio.2025.101493)

TRITC

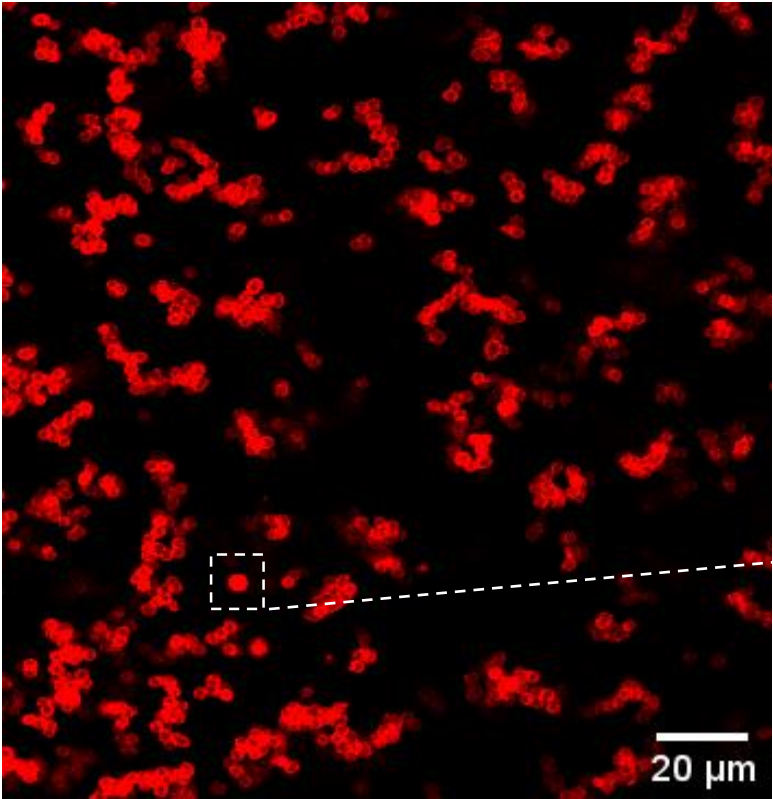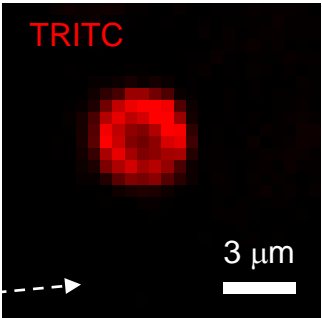

Supplement: Fig. S1 — A suspension of the Parg/DS-composed microcapsules before administration to neuronal cultures in vitro. The image is taken with two-photon excitation (2 PE) microscopy at λx2PE = 960 nm. [file mmc1.pdf]

Microcapsules: 4–5 hours

TL / TRITC

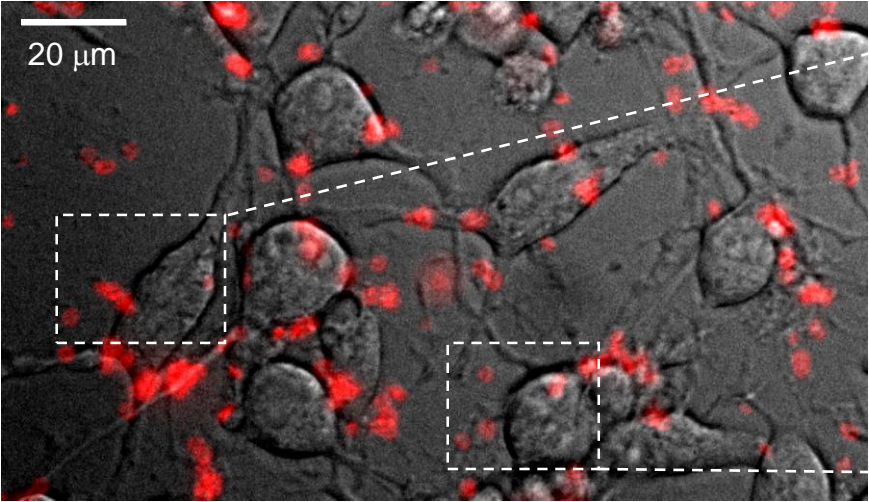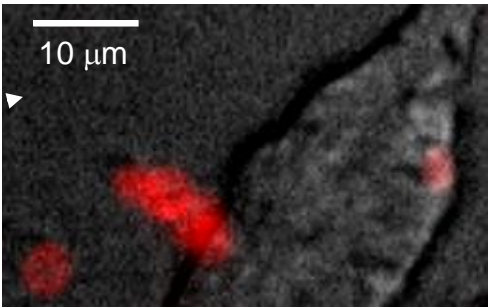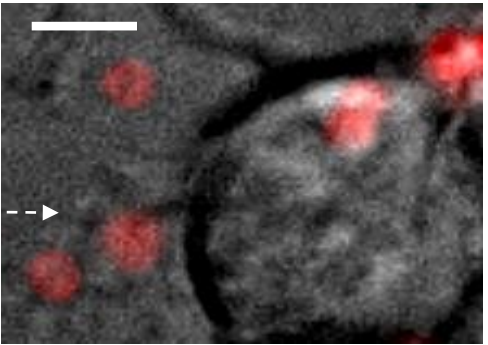

Microcapsules: 24 hours

TL / TRITC

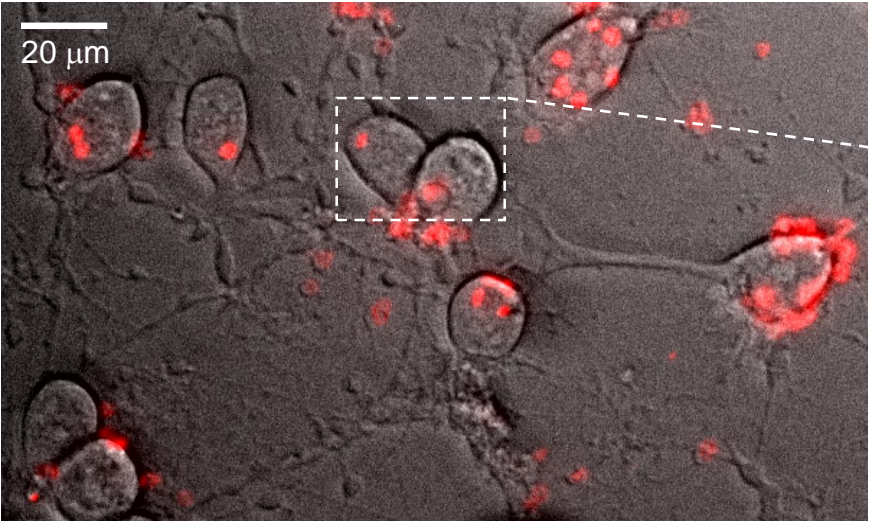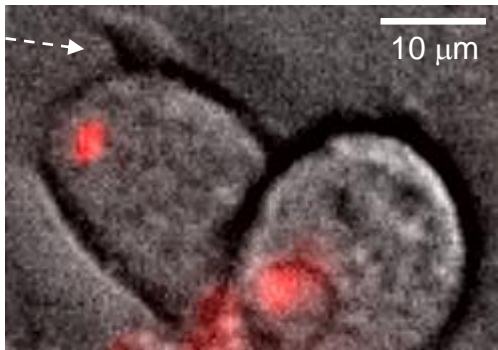

Supplement: Fig. S2 — Differentiating N2A cells of neuronal phenotype effectively uptake polyelectrolyte PArg/DS-based microcapsules. Images show N2A cells after 4–5 h of incubation (top images) and 24 h of incubation with a dispersed suspension of microcapsules. Right images, enlarged cells (dotted square) with microcapsules inside. Scale bar: 10 μm. Merged red channel (TRITC, microcapsule shell) and transmitted light (TL) channel. [file mmc2.pdf]

Microcapsules: 24 hours

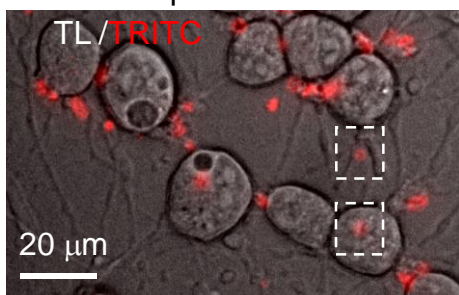

48 hours

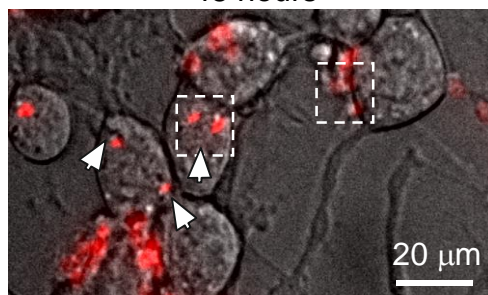

72 hours

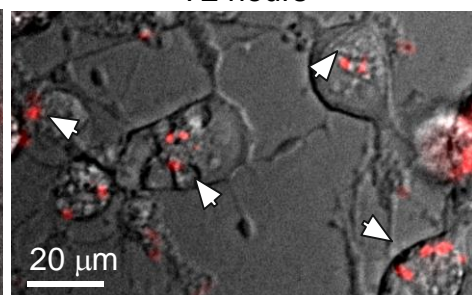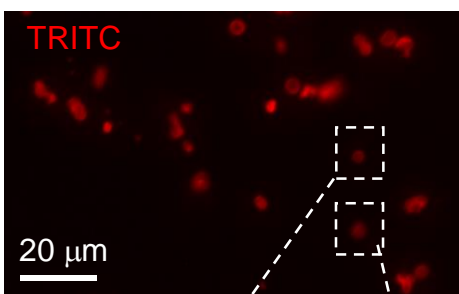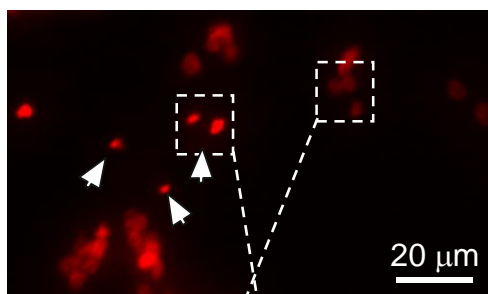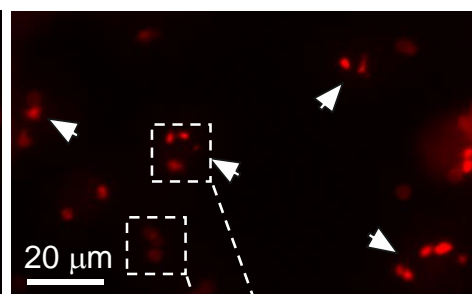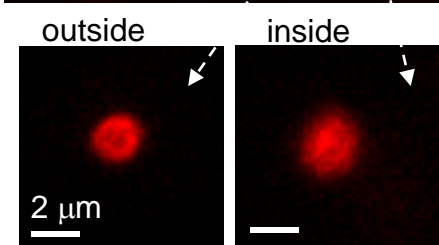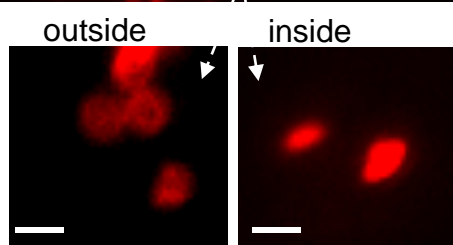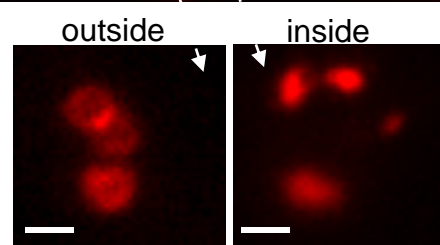

Supplement: Fig. S3 — Intracellular biodegradation of polyelectrolyte PArg/DS microcapsules by N2A cells of neuronal phenotype. Images of differentiated N2A cells after 24 h (left panel), 48 h (middle panel) and 72 h of incubation (right panel) with a suspension of PArg/DS microcapsules. The bottom row shows enlarged microcapsules (dotted square in images above) outside and inside the cells. Scale bar: 2 μm. The top row shows the merged red channel (TRITC, microcapsule shell) and transmitted light (TL) channel. [file mmc3.pdf]

1 d incubation

Z: 3  $\mu\text{m}$

TL / DIO / TRITC

30  $\mu\text{m}$

Z: 6  $\mu\text{m}$

30  $\mu\text{m}$

Z: 10  $\mu\text{m}$

30  $\mu\text{m}$

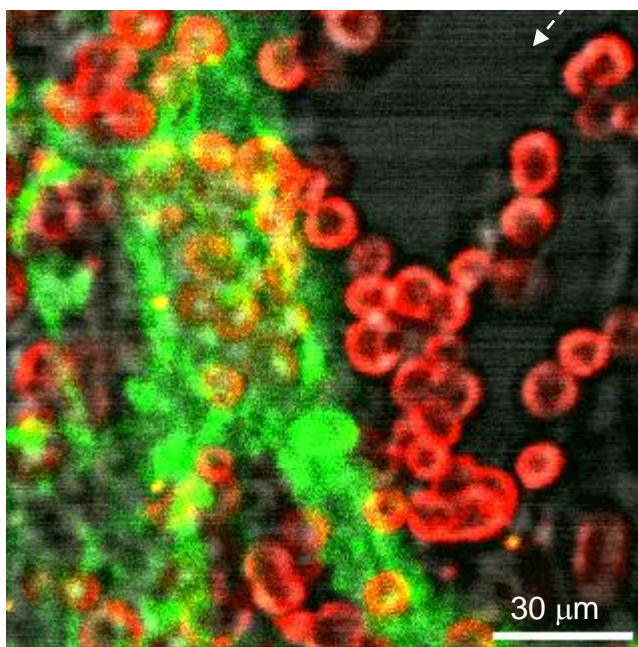

Supplement: Fig. S4 — Microcapsule uptake by hippocampal neurons in vitro. Two-photon excitation (2 PE) images of cultured hippocampal neurons (34 DIV) supplemented with a suspension of PArg/DS microcapsules across various focal planes as indicated. The lower image shows an enlarged snapshot of the indicated area (white dotted square in the top image). Images are merged transmitted light (TL), green fluorescent channel (DiO, plasma membrane staining) and red channel (TRITC, microcapsule shell). [file mmc4.pdf]

Microcapsules: 8 hours

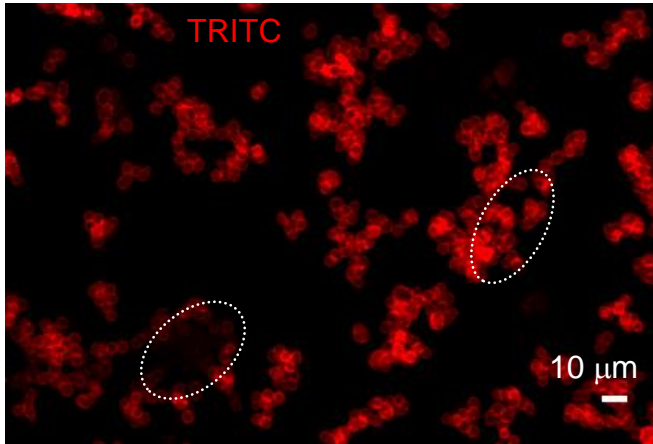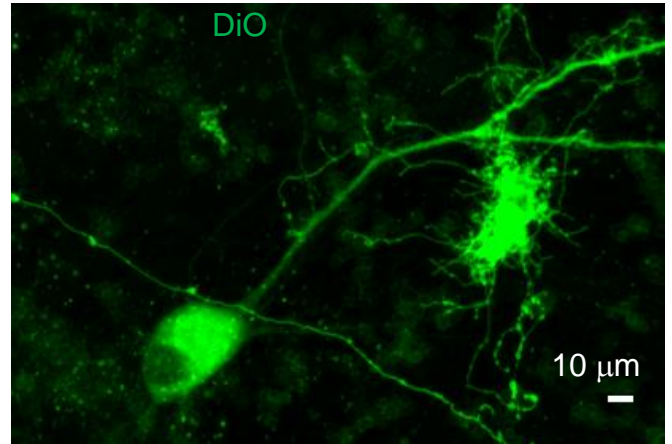

Supplement: Fig. S5 — Microcapsule uptake by hippocampal neuronal-astrocytic co-cultures. Two-photon excitation (2 PE) images of hippocampal neuronal-astrocytic co-cultures supplemented with a suspension of microcapsules (8 h of incubation in vitro). The left image is the red fluorescent channel (TRITC fluorescence). Right image, the green channel (DiO, the plasma membrane staining). [file mmc5.pdf]

**A**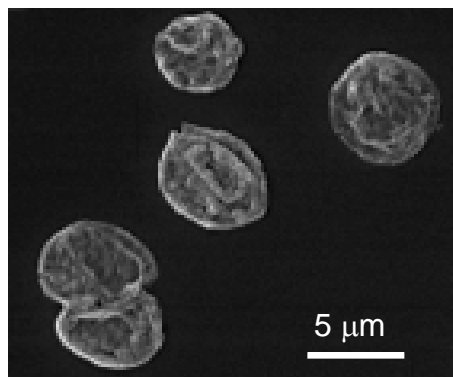**B**

1 d incubation

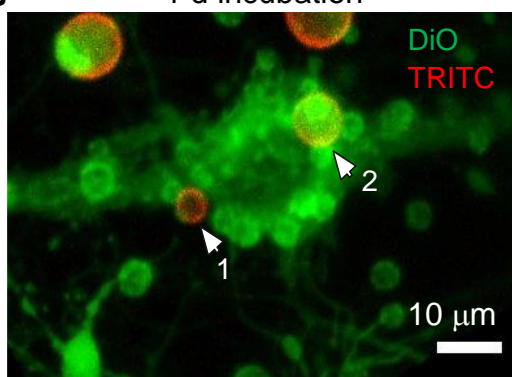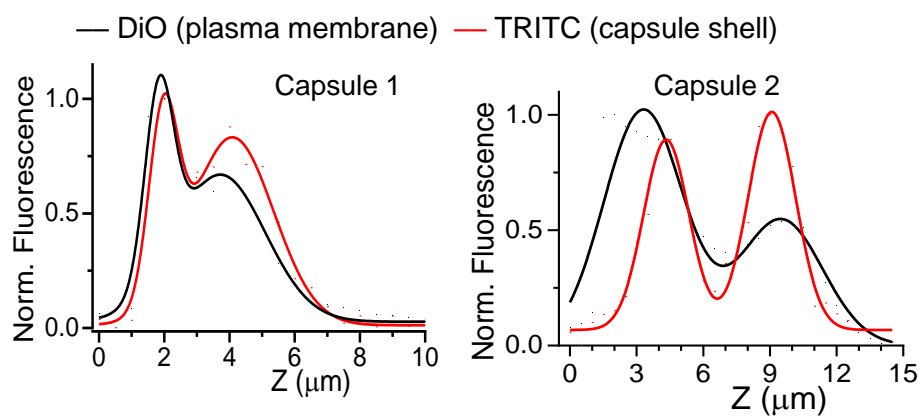**C**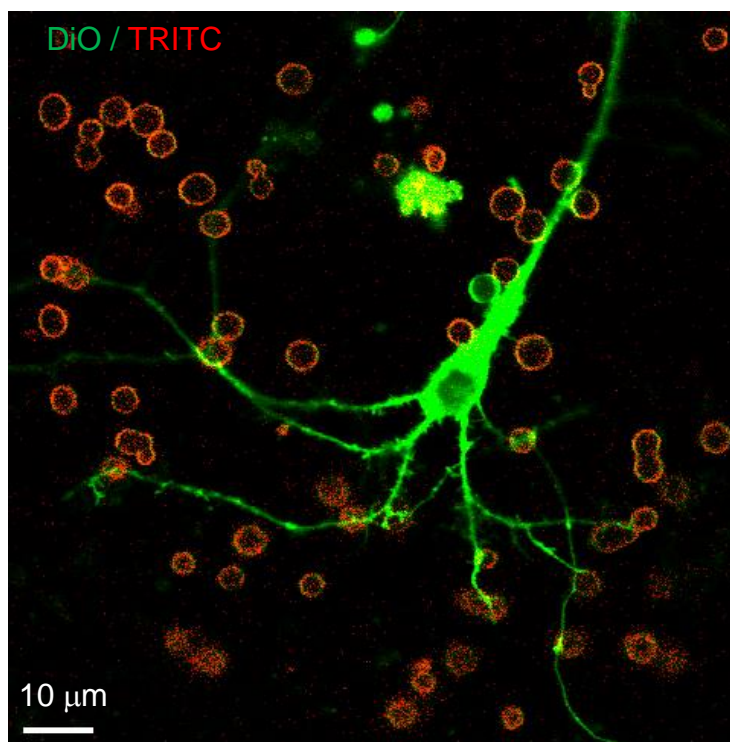

Supplement: Fig. S6 — Neuronal uptake of larger-sized microcapsules. (A) SEM image of the PArg/DS-based microcapsules fabricated with a larger diameter (∼5 μm) (B) 2 PE image of a hippocampal neuron after 1 d incubation with larger diameter PArg/DS microcapsules (top) and Z-profiles (lower plots) for the microcapsule's shell (TRITC, red channel) and plasma membrane fluorescence (DiO, green channel) for two microcapsules (1 and 2) indicated on the upper image. (C) 2 PE image of a hippocampal neuron after 1 d incubation with large-sized PArg/DS microcapsules shows no intracellular microcapsules but co-localisation with neuronal structures. [file mmc6.pdf]

Z: 5  $\mu\text{m}$

Microcapsules + Dextran

Z: 10  $\mu\text{m}$

TL / TRITC / Dextran

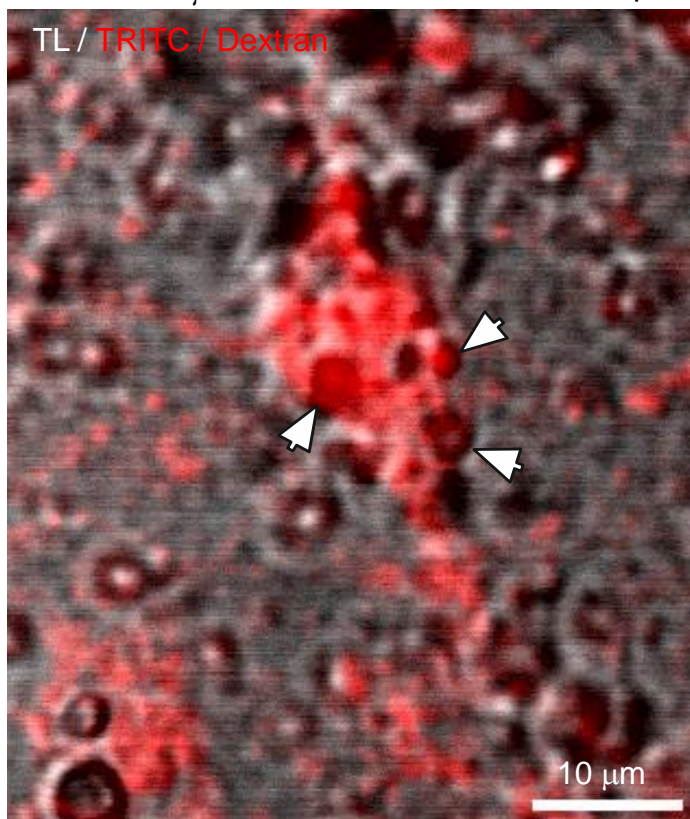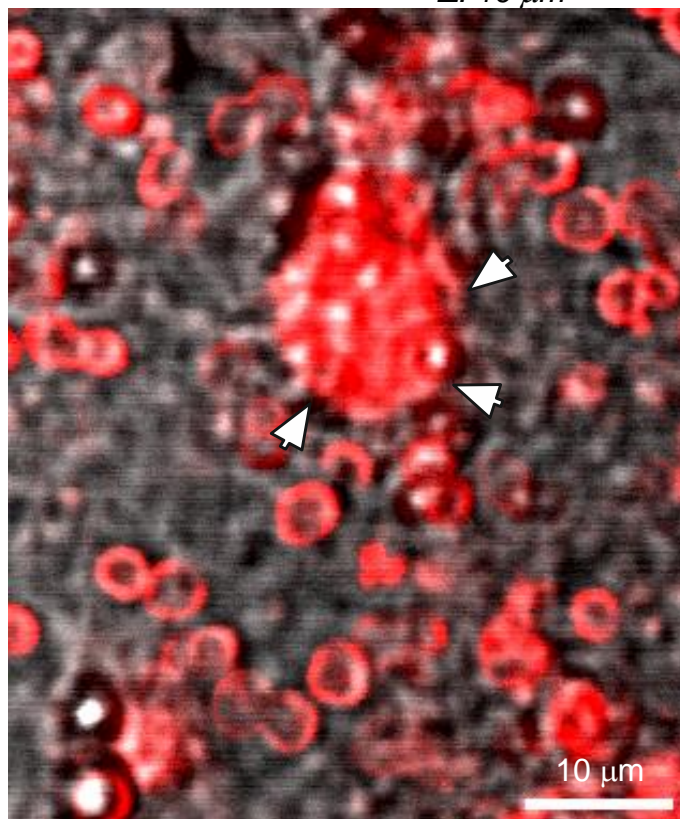

Supplement: Fig. S7 — Microcapsule internalisation relates to macropinocytosis by hippocampal neurons. Two-photon excitation (2 PE) images of hippocampal neurons supplemented with a suspension of PArg/DS microcapsules in the presence of high molecular weight Dextran Texas Red (8 h of incubation in vitro). Images are merged transmitted light (TL) and red fluorescent channels (TRITC and dextran fluorescence) at different Z-depth, as noted. [file mmc7.pdf]
